# Clinical utility of pharmacogenetic testing in children and adolescents with severe mental disorders

**DOI:** 10.1007/s00702-018-1882-4

**Published:** 2018-04-06

**Authors:** Hilario Blasco-Fontecilla

**Affiliations:** 1 Department of Psychiatry, Segovia de Arana Health Research Institute (IDIPHISA)-Puerta de Hierro University Hospital, Avenida Manuel de Falla s/n, Majadahonda, Madrid, Spain; 2 Autonoma University, Madrid, Spain; 3 Biomedical Research Centre in Mental Health Net (CIBERSAM), Madrid, Spain

**Keywords:** Pharmacogenetic decision support tool, Pharmacogenetic testing, Mental disorder, Children, Personalized psychiatry, Polypharmacy

## Abstract

This is a retrospective cohort study of 20 children and adolescents to evaluate the clinical utility of a pharmacogenetic decision support tool. Twenty children and adolescents underwent pharmacogenetic testing between June 2014 and May 2017. All children and adolescents were evaluated at Puerta de Hierro University Hospital-Majadahonda (Madrid, Spain). We report the proportion of patients achieving clinical improvement, amelioration of side effects, and changes in number of drugs. Data normality was assessed with the Shapiro–Wilk test, and changes of pre- and post-pharmacogenetic testing were analyzed with the Wilcoxon test for paired samples. A two-sided *p* value threshold of 0.05 was considered for significance. Pharmacogenetic testing helped to improve the clinical outcome as measured by the Clinical Global Impressions (CGI) Scale in virtually all children (95%; 19 out of 20 children). The CGI improvement (CGI-I) was 2 (0.79) (range 1–4), 2.1 (0.56) (range 1–3), and 1.9 (0.99) (range 1–4) in foster and non-foster care children, respectively. Pharmacogenetic testing also helped to reduce the number of children using polypharmacy (from 65 to 45%), the mean number of drugs per children (from 3.3 to 2.4 drugs, *p* = 0.017), and self-reported relevant side effects (*p* = 0.006). Pharmacogenetic testing helped to improve the clinical outcome, and to reduce polypharmacy and the number of drugs used in children and adolescents with severe mental disorders. More evidence using robust (i.e., clinical trials) independent studies is required to properly determine the clinical utility and cost-effectiveness of pharmacogenetic testing tools in children and adolescents with mental disorders.

**Electronic supplementary material:**

The online version of this article (10.1007/s00702-018-1882-4) contains supplementary material, which is available to authorized users.

## Background

An increasingly large proportion of the worldwide population lives with a mental disorder. The worldwide prevalence of mental disorders is 20% in adult populations (Kessler et al. 2009; Steel et al. 2014), and 13.4% (95% CI 11.3–15.9) in children and adolescents (Polanczyk et al. 2015). Medications are frequently used to treat mental disorders, but “choosing the right medication for each patient is challenging” (Health Quality Ontario 2017). Indeed, a great proportion of patients discontinue medication because of lack of response or adverse effects (Health Quality Ontario 2017). For instance, the treatment for major depressive disorder (MDD) does not achieve remission in approximately 50% of patients following two drug trials (Rush 2007). On the other hand, medications such as antipsychotics can produce extra-pyramidal symptoms, metabolic syndrome, and elevation of blood prolactin, among others (Leucht et al. 2013).

Given the high proportion of lack of response and the patient’s need of achieving quick symptom relief, polypharmacy has become a frequent practice (Mrazek 2010). Even if support for polypharmacy is scarce (Stahl 2002), polypharmacy and off-label use of psychotropic drugs are frequent in youth with mental disorders and have become a matter of concern worldwide (Olashore and Rukewe 2017). This is probably a consequence of the reality that the process of decision-making about the best drug choice for each patient still remains an empirical “trial-and-error process” (Mrazek 2010).

In this context, pharmacogenomic and pharmacogenetic (PGx) testing may help to guide decision-making regarding the best personalized prescription for each patient (Arandjelovic et al. 2017; Bousman and Hopwood 2016) by increasing the rate of response, lowering the rate of side effects (Haga and LaPointe 2013), and reducing psychiatric healthcare costs (Plothner et al. 2016). Children and adolescents with mental disorders should benefit from the pharmacogenomic promise to use “safer and more effective drugs” (Freund and Clayton 2003). But even if the translation of pharmacogenomics to individualized psychiatry for children and adolescents has accelerated (Wehry et al. 2018), PGx testing is not routine, and proper study of clinical utility is being undertaken slowly (Bousman et al. 2017a).

This is a retrospective cohort study of 20 children and adolescents who underwent PGx testing using a particular pharmacogenetic decision support tool. Preliminary reports using this tool in adult samples are encouraging (Espadaler et al. 2016; Perez et al. 2017), but there are no publications in youth populations. The aim of the present study was to describe the clinical utility of Neuropharmagen for children with severe mental disorders under real-world conditions. The clinical utility was described in terms of clinical outcome, decrease of either polypharmacy or the number of drugs used, and reduction in side effects.

## Materials and methods

### Study design

This is a retrospective cohort study of children and adolescents with severe mental disorders who received treatment and underwent PGx testing with Neuropharmagen at Puerta de Hierro University Hospital-Majadahonda (HUPH-M, Madrid, Spain) between June 2014 and May 2017. Neuropharmagen is a pharmacogenomic-based precision medicine platform developed by AB-Biotics SA (Barcelona, Spain) to assist clinicians in drug selection (Perez et al. 2017). After gaining the approval of the ethics committee, we prepared a chart including the following information, which was extracted from the clinical files of each patient: (1) Diagnostic and Statistical Manual of Mental Disorders-Fourth Edition (DSM-IV) diagnoses and clinical severity as measured by the Clinical Global Impression Scale-Severity Component (CGI-S) (Busner and Targum 2007), (2) the main reasons for PGx testing, which were classified into three categories (poor clinical response, adverse events reported spontaneously, and the need by either the clinician or the parents/guardians of the child to confirm that the drug regimen was the best available choice for the patient), (3) pre- and post-PGx testing drug regime, (4) PGx testing recommendation, (5) clinical outcome (as measured by the improvement component of the CGI, CGI-I) (Busner and Targum 2007), and (6) improvement (if any) of the self-reported relevant side effects collected using a non-validated questionnaire including neurologic side effects (dystonia, tremor, akathisia, seizures, and tics), lost/gained weight, accommodation disturbances, dry mouth, excessive sedation, palpitations/tachycardia, nausea/vomiting, headache, galactorrhoea/amenorrhoea, excessive/reduced duration of sleep, and “others” side effects.

### Study sample

We included 20 patients (10 children and adolescents living in residential foster care; and 10 children and adolescents who were not) evaluated at HUPH-M from June 1, 2014 to May 25, 2017. All patients were 17 years of age or younger and were diagnosed according to the Diagnostic and Statistical Manual of Mental Disorders-Fourth Edition (DSM-IV).

### Genotyping and reporting of test results

All patients provided a saliva sample for DNA extraction and genotyping. The way DNA is usually extracted from saliva samples, how the genotyping of single-nucleotide polymorphisms (SNPs) is performed, and the PGx testing interpretative report of this tool is reported elsewhere (Perez et al. 2017). Basically, the current version of this pharmacogenetic decision support tool report provides three types of information: (1) pharmacogenomic information for 50 drugs, including antipsychotics, antidepressants, mood stabilizers, and other CNS drugs, derived from the analysis of SNPs in 25 genes associated with drug efficacy, metabolism, or specific adverse effects (e.g., extra-pyramidal symptoms or metabolic syndrome); (2) data on pharmacological interactions; and (3) information on lifestyle influences and specific clinical conditions. The information is accessible through a Web-based computer-aided system.

### Change of medication after PGx testing

The information provided by PGx testing was always considered when making a medical decision, including putative changes in the drug regime. Choosing the right medication for each child was made as usual, including all available information (clinical diagnosis and symptoms, potential side effects, efficacy of drugs, etc.) but incorporating the information provided by the pharmacogenetic decision support tool.

### Ethics

All patients and their legal guardians had provided written informed consent for PGx testing. The study was approved by the Agencia Española de Medicamentos y Productos Sanitarios (AEMPS). The study protocol was approved by the IRB of the Puerta de Hierro University Hospital-Majadahonda (Madrid, Spain) (reference number 17.17; October 9, 2017). The study complied with the Helsinki Declaration.

### Statistics

Data normality was assessed with the Shapiro–Wilk test. Because of the lack of normality detected, changes in pre- and post-PGx testing were analyzed with the Wilcoxon non-parametric test for paired samples, and correlation was measured with the Spearman non-parametric test. A two-sided *p* value threshold of 0.05 was considered for significance.

## Results

### Descriptive results

Characteristics of the 20 patients are shown in Table 1 [see also Table 1-Supplementary Material (SM) (foster care children) and Table 2-SM (non-foster care children) for further information].

**Table 1:** Main characteristics of the sample

	All children	Foster care children	Non-foster care children	Significance
Age ( SD)	14.6 ± 1.5	14 ± 1.49	15.2 ± 1.39	*t* = − 1.857, *df* = 18, *p* = 0.080
Gender (female, %)	11 (55%)	8 (80%)	3 (30%)	FET *p* = 0.070
Main axis I diagnose	Autistic disorder (299.00) (25%) Attention-deficit hyperactivity disorder, combined subtype (314.01) (25%)	Autistic disorder (299.00) (50%)	Major depressive disorder (MDD), single episode (severe without psychotic features, 296.23) (40%)	*χ*^2^ = 13.200, *df* = 6, *p* = 0.040
*N* of concomitant drugs pre-PGx testing (mSD)	3.3 ± 1.86	4 ± 2.26	2.6 ± 1.07	*t* = 1.769, *df* = 18, *p* = 0.094
*N* of concomitant drugs post- PGx testing (mSD)	2.4 ± 0.93	2.7 ± 0.94	2 ± 0.81	*t* = 1.769, *df* = 18, *p* = 0.094
CGI-S (pre-)	5.6 ± 0.98	6.2 ± 0.63	5.1 ± 0.99	*t* = 2.952, *df* = 18, *p* = 0.009
CGI-S (post-)	3.8 ± 1.03	4.1 ± 0.87	3.6 ± 1.17	*t* = 1.080, *df* = 18, *p* = 0.295

*FET* Fisher’s exact test

Poor clinical response was the most frequent reason for PGx testing (19 out of the 20 cases). Furthermore, before PGx testing, 13 (65%) children [7 (70%) and 6 (60%) of the foster care and non-foster care children, respectively] were on polypharmacy, as defined by the use of three or more concomitant psychotropic medications (Fontanella et al. 2014). Finally, self-reported clinically significant side effects were the second most frequently reported reason for using PGx testing (*n* = 9, 45%). Five (50%) and four (40%) of the foster care and non-foster care children spontaneously reported at least one side effect, respectively. Side effects included weight increase (*n* = 3), neck dystonia (*n* = 2), akathisia (*n* = 2), neck dystonia and akathisia (*n* = 2), headaches (*n* = 1), and excessive sedation (*n* = 1).

### Clinical outcomes

**Fig. 1:**
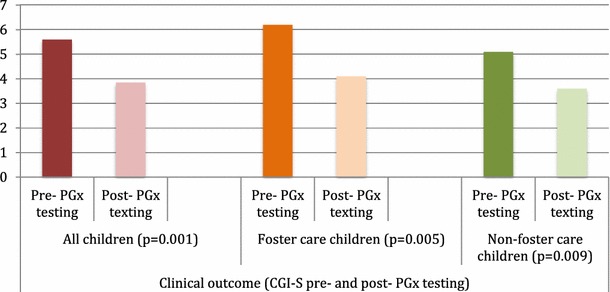
Clinical outcome as measured by the CGI pre- and post-PGx testing

We found clinical improvement as measured by the CGI-I in virtually all children (95%; 19 out of 20 children) after the use of PGx testing. The average CGI-I was 2 (0.79) (range 1–4) in the study population: 2.1 (0.56) (range 1–3) and 1.9 (0.99) (range 1–4) in foster and non-foster care children, respectively. The CGI-S post-PGx testing (subtracting CGI-I to the basal CGI-S) was 3.8 (1.03) (range 0–6) (*p* < 0.001) (foster care children: 4.10 (0.87) (range 3–5) (*p* = 0.005); and non-foster care children: 3.6 (1.17) (range 0–6) (*p* = 0.009) (see Fig. 1).

### Polypharmacy

**Fig. 2:**
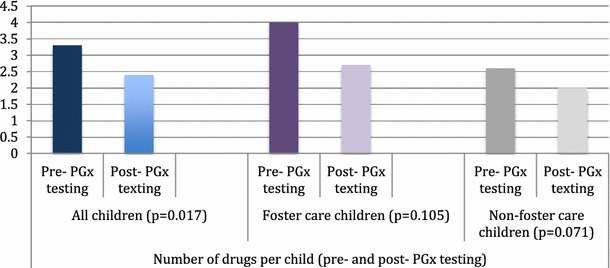
Reduction in the number of drugs per child pre- and post- PGx testing). Foster and non-foster are shown separately to illustrate that the reduction is found in both groups. The global reduction in number of drugs was statistically significant (*p* = 0.017). However, the number of subjects in each subgroup was too small for the reduction to reach statistical significance within each subgroup (*p* = 0.105 and *p* = 0.071 in foster and non-foster, respectively)

After PGx testing, 9 (45%) children [6 (60%) and 3 (30%) of the foster care and non-foster care children, respectively] were on polypharmacy. In other words, there was a reduction of polypharmacy in 20% of the children (10% of foster care children and 30% of non-foster care children). There was also a reduction in the mean number of drugs per children, as they were on 2.4 ± 0.9 drugs on average (47 drugs/20 children: 2.7 drugs per foster care child and 2 drugs per non-foster care child), the change being statistically significant (*p* = 0.017) when the data were treated as a group (see Fig. 2). Of note, said reduction was strongly correlated to the number of drugs at baseline (Spearman *r* = 0.81, *p* < 0.001), indicating that patients taking a higher number of drugs were those benefitting from a larger reduction.

### Adverse events (side effects)

In all cases, the reported side effects were no longer a matter of concern after the change in the treatment regimen following pharmacogenetic testing. Given that, at baseline, there were 0.6 ± 0.7 (range 0–2) relevant adverse effects per participant; their complete disappearance post- pharmacogenetic testing was statistically significant (*p* = 0.006).

## Discussion

The present naturalistic retrospective descriptive cohort study of 20 children suggests that pharmacogenetic testing may have clinical utility in improving clinical outcomes, reducing polypharmacy, reducing the number of drugs used per child, and reducing side effects in children with severe mental disorders. Two previous studies (one retrospective naturalistic multicenter study and a randomized double-blind clinical trial) in adults with mental disorders further support the clinical utility of this particular pharmacogenetic decision support tool (Espadaler et al. 2016; Perez et al. 2017). This is in keeping with the recent literature suggesting the clinical utility of other pharmacogenetic tools such as Amplichip (Chau and Thomas 2015). Furthermore, two recent reviews, one of them focused on child psychiatry, suggest that even if the improvement in health outcomes and the cost-effectiveness of pharmacogenomics are not consistently replicated, PGx testing appears to be a promising tool that might help in predicting treatment response and adverse events (Rosenblat et al. 2017; Wehry et al. 2018).

The most relevant finding of the present study is that pharmacogenetic testing contributed to improved clinical outcomes as measured by the CGI-I in nearly all children. The median CGI-I score of 2 (much improved) (Busner and Targum 2007) reflects that, in some cases, the clinical improvement was dramatic, particularly in foster care children. In a recent systematic review of clinical trials and cost-effectiveness studies of PGx testing on the clinical outcome of MDD, the authors concluded that clear-cut demonstration of improved health outcomes and cost-effectiveness of pharmacogenomics were not yet supported with replicated evidence; however, they recognized that some studies have reported promising results for the clinical utility of PGx testing (Rosenblat et al. 2017). In another review of PGx testing in child and adolescent psychiatry, the authors concluded that PGx testing might help in predicting treatment response and adverse events, as well as medication selection in children and adolescents with depressive disorders, anxiety disorders, and ADHD (Wehry et al. 2018). Furthermore, in a case report using the Genecept assay testing (Genomind, Chalfont, PA, USA), the authors reported that PGx testing helped in prescribing the most effective medication, thus illustrating “how pharmacogenetics and psychiatry can potentially interface to provide more informed decision-making regarding use of psychotropic medications” (Smith et al. 2015).

We also found a notable reduction in polypharmacy and the number of drugs per child, the effect being statistically significant. Moreover, the reduction effect was larger in those patients taking more drugs. Clinicians usually turn to polypharmacy in a desperate intent to find the “right combination” (Mrazek 2010) and control psychiatric symptoms. Furthermore, the pressure to promptly resolve psychiatric symptoms makes pharmacological options more attractive, thus favoring polypharmacy (Diaz-Caneja et al. 2014; Olashore and Rukewe 2017). However, polypharmacy increases the risk of drug–drug interactions, side effects, and non-compliance (Olashore and Rukewe 2017; Stahl 2002). Polypharmacy was particularly worrisome in our sample of foster care children. This was not surprising given that foster care children displayed more severe mental disorders. Foster care children are more likely to use mental health services than non-foster care children (Halfon et al. 1992), and the use of polypharmacy is frequent. In a study of 240 youths in foster care, 61% were taking two or more prescribed drugs (Brenner et al. 2014), which is comparable with the 80% that we reported here. Furthermore, a few non-pharmacological options have been successful in reducing behavioral problems in foster care youth. For instance, Multidimensional Treatment Foster Care for Preschoolers was less efficient than regular foster care in diminishing the severity of externalizing problems in a recent study (Jonkman et al. 2017).

The reduction of polypharmacy was accompanied with the resolution of all clinically significant side effects. This is not surprising, as polypharmacy is related to overmedication and side effects (Mrazek 2010; Olashore and Rukewe 2017). This is important, because children are particularly vulnerable to side effects, particularly extra-pyramidal symptoms, when using antipsychotics (Garcia-Amador et al. 2015). Our results are in keeping with a 12-week double-blind randomized controlled trial in 316 adult patients with MDD using the same pharmacogenetic test. The authors reported that the use of PGx testing was associated with an increased likelihood of achieving better tolerability (frequency, intensity, and burden of side effects) (Perez et al. 2017). This finding is particularly relevant for our children with autism spectrum disorders, as they are “less equipped to express potential side effects of medications or have full remission of symptoms with medication” (Bose-Brill et al. 2017).

Strengths of the present study include: (1) the naturalistic, real-world clinical practice design and (2) a population of particular interest: children and adolescents with severe mental disorders. To our knowledge, there are no previous reports of clinical use of any particular PGx test in children and adolescents except for a two-case report study using Neuropharmagen (De Crescenzo et al. 2016) and a single case report using Genecept assay testing (Smith et al. 2015). Limitations include: (1) a small sample size; (2) the particularly severe profile, particularly of foster care children, which are not representative of the “regular” children followed-up at mental health centers; (3) the lack of clinical scales apart from CGS-S and CGS-I; (4) the lack of a proper control group; (5) the use of one of the potential definitions of polypharmacy (Chen et al. 2011; Fontanella et al. 2014) and clinical utility (Bousman and Hopwood 2016; de Leon 2016); and, finally, (6) our lack of opportunity to conduct a cost-effectiveness analysis. All these limitations put together imply that our data cannot be generalized to all children and adolescents with mental disorders. Additional studies with more detailed clinical scales, larger populations, and a randomized double-blind design (i.e., clinical trials) are required to confirm the clinical utility of PGx testing in the general population of children and adolescents with mental health disorders.

## Conclusion

Preliminary reports of different pharmacogenetic decision support tools are promising (Espadaler et al. 2016; Smith et al. 2015; Chau and Thomas 2015). Our study is in keeping with these studies but in a youth population, and suggests that PGx testing might help clinicians in the process of decision-making about the best drug choice for a particular child diagnosed with a mental disorder. However, more evidence concerning the clinical utility of PGx testing in youth is warranted. All pharmacogenetic decision support tools will probably have to face similar problems. For instance, Amplichip still must compile evidence regarding genotype accuracy, predictions of phenotypes from genotypes, and the translation of genotype-to-phenotype predictions into clinical utility (Chau and Thomas 2015). Moreover, the body of evidence regarding the GeneSight test is very low and mostly limited to depression (Health Quality Ontario 2017). Thus, more evidence is required to properly determine the clinical utility and cost-effectiveness of PGx testing in children and adolescents, and whether some patient profiles are more likely to benefit than others.

Some have considered “the moral treatment of mental patients, electro-convulsive therapy (ECT), and psychotropic medications”, along with addressing the comorbidities of mental illnesses with chronic physical illnesses as the first, second, third, and fourth revolutions in psychiatry (Gautam 2010). Personalized psychiatry may constitute the fifth psychiatric revolution. To achieve this aim, personalized psychiatry must demonstrate its clinical utility by mounting more evidence and becoming part of the clinical routine, and this would not be easy. A roadmap for “pharmacogenetic tests to be incorporated by prescribers into long-term practice” has recently been published (de Leon 2016; de Leon and Spina 2016). Furthermore, the universal adoption of the different PGx tools should rely on independent evaluations (Bousman et al. 2017b). In any case, as warned by Bousman et al. (2018), PGx testing will never provide “definitive prescribing advice for psychiatric drugs” or substitute for a good clinician. PGx tests just provide additional information for clinicians to assist them in choosing the right drug within the biopsychosocial context of a particular patient (Arandjelovic et al. 2017; Bousman et al. 2018) who is probably not diagnosed with a disease but rather a syndrome (de Leon 2016).

## Electronic supplementary material

Below is the link to the electronic supplementary material.

Supplementary material 1 (DOCX 30 kb)

## Abbreviations

ADHD: Attention deficit hyperactivity disorder
CGI Scale: The Clinical Global Impressions
CGI-S: The Clinical Global Impressions-Severity
CGI-I: The Clinical Global Impressions-Improvement
ECT: Electro convulsive therapy
MDD: Major depressive disorder
PGx: Testing pharmacogenetic testing
