# Supplementary material for: Clinical utility of pharmacogenetic testing in children and adolescents with severe mental disorders
Source: J Neural Transm (Vienna). 2018 Apr 6;126(1):101–7. doi: 10.1007/s00702-018-1882-4 (PMC6373261; doi:10.1007/s00702-018-1882-4)
Supplement: Supplementary file 1 — Supplementary material 1 (DOCX 30 kb) [file 702_2018_1882_MOESM1_ESM.docx]

**Table 1-SM. Characteristics of the sample of children and adolescents in foster care.**

| **Case** | **Age (years)/ Sex** | **(M) Main diagnose(s) (DSM-IV)**  **(R) Reason for PGx testing (PCR/AE/CO*)** | **(D) pre-PGx testing Drugs (mg per day)**  **(N) Number of concomitant drugs** | **(D) post-PGx testing Drugs (mg per day)**  **(N) Number of concomitant drugs** | **(1) PGx testing recommendation**  **(2) Clinical outcome (CGI-S and CGI-I)**  **(3) Reduction in side effects (Yes/No/NA)** |
| --- | --- | --- | --- | --- | --- |
| 1 | 16M | (M) 1) Attention-Deficit Hyperactivity Disorder, combined subtype (314.01)  2) Conduct Disorder, childhood onset (312.81)  (R) PCR (severe aggressive behaviors) and AE (akathisia, neck dystonia) | (D) - Atomoxetine (Strattera®) (80 mg)  - Long-acting methylphenidate (Medikinet®) (20 mg)  - Risperid4one (Risperdal®) (4 mg)  - Olanzapine (Zyprexa®) (20 mg)  - Melatonin (Circadin®) (2 mg)  - Propranolol (Sumial®) (10 mg)  - Biperiden (Akineton®) (4 mg)  - Lorazepam (3 mg)  (N) 8 | (D) - Long-acting methylphenidate Medikinet® (50 mg)  - Quetiapine (Seroquel®) (300 mg)  (N) 2 | (1) More likely to respond to methylphenidate (ADRA2A); antipsychotics with standard response: quetiapine and ziprasidone  (2) CGI-S=6; CGI-I=1  (3) Yes |
| 2 | 12F | (M) Bipolar I Disorder, most recent episode manic (severe without psychotic features, 296.43)  (R) PCR (impossibility of controlling manic symptoms) and AE (neck dystonia using low doses of risperidone, and weight increase using olanzapine) | (D) - Risperidone (Risperdal®) (1 mg)  - Olanzapine (Zyprexa®) (20 mg)  - Valproic acid (Depakine Crono®) (1000 mg)  - Clonazepam (Rivotril®) (6 mg)  - Carbamazepine (Tegretol®) (600 mg)  (N) 5 | (D) - Lithium (Plenur®) (1200 mg)  - Oxcarbazepine (Trileptal®) (1200 mg)  (N) 2 | (1) More likely to respond to lithium (CACNG2), resistance to antiepileptics [valproic acid, clonazepam and carbamazepine (ABCB1)]; increased metabolization of carbamazepine (EPHX1); standard response to oxcarbazepine  (2) CGI-S=7; CGI-I=2  (3) Yes |
| 3 | 14M | (M) Autistic Disorder (299.00)  (R) PCR (aggression) and AE (akathisia) | (D) - Haloperidol (6 mg)  - Oxcarbazepine (Trileptal®) (1500 mg)  - Lithium (Plenur®) (1000 mg)  - Biperiden (Akineton®) (8 mg)  - Clopixol (20 mg)  - Clonidine (Catapresan®) (2 mg)  (N) 6 | (D)- Haloperidol (8 mg) - Oxcarbazepine (Trileptal®) (1200 mg)  - Lithium (Plenur®) (1200 mg)  (N) 3 | (1) More likely to respond to lithium (CACNG2) and less likely to develop extrapyramidal symptoms with haloperidol (AKT1-DDIT4-FCHSD1-RPTOR)  (2) CGI-I=6; CGI-S=3  (3) Yes |
| 4 | 13M | (M) Autistic Disorder (299.00)  Epilepsy  (R) PCR (worsening of echolalia and stereotyped behavior) | (D)- Long-acting methylphenidate (Concerta ®) (54 mgs)  - Risperidone (Risperdal®) (4 mgs) - Oxcarbazepine (Trileptal®) (750 mgs)  - Clobazam (Noiafren®) (30 mgs)  (N) 4 | (D)- Long-acting methylphenidate (Concerta ®) (54 mg)  - Risperidone (Risperdal®) (4 mgs) - Oxcarbazepine (Trileptal®) (1200 mg)  - Perampanel (Fycompa®) (12 mg)  (N) 4 | (1) More likely to respond to methylphenidate (COMT, LPHN3) and less likely to develop extrapyramidal symptoms with risperidone (AKT1-DDIT4-FCHSD1-RPTOR); lower metabolization of risperidone (CYP2D6)  (2) CGI-S=6; CGI-I=3  (3) Yes |
| 5 | 15F | (M) Moderate mental retardation (318.0)  (R) PCR (Poor behavior control) | (D) - Risperidone (Risperdal®) (0.5 mg)  - Oxcarbazepine (Trileptal®) (200 mg)  - Sertraline (Besitran®) (100 mg)  (N) 3 | (D) - Paliperidone (Invega®) (3 mg)  - Quetiapine (Seroquel®) (600 mg)  - Sertraline (Besitran®) (200 mg)  (N) 3 | (1) Less likely to develop extrapyramidal symptoms with either risperidone or paliperidone (AKT1-DDIT4-FCHSD1-RPTOR); lower metabolization of risperidone (CYP2D6)  (2) CGI-S=6; CGI-S=2  (3) NA |
| 6 | 12M | (M) - ADHD, combined subtype (314.01)  - Conduct Disorder, childhood onset (312.81)  (R) PCR (Antisocial behaviors, and depressive symptoms, particularly insomnia) | (D) - Long-acting methylphenidate  (Concerta ®) (54 mg)  - Risperidone (Risperdal®) (4 mg) - Oxcarbazepine (Trileptal®) (600 mg)  - Chlorpromazine (Sinogan®) (100 mg)  - Biperiden (Akineton®) (4 mg)  - Melatonin (Circadin®) (2mg)  (N) 5 | (D) - Long-acting methylphenidate  (Concerta ®) (54 mg)  - Paliperidone (Invega®) (3 mg)  - Fluvoxamine (Dumirox®) (100 mg)  (N) 3 | (1) More likely to respond to methylphenidate (ADRA2A) and fluvoxamine (HTR1A); less likely to develop extrapyramidal symptoms with paliperidone (AKT1-DDIT4-FCHSD1-RPTOR);  (2) CGI-S=5; CGI-I=2  (3) Yes |
| 7 | 15M | (M) Autistic Disorder (299.00)  (R) PCR (Incontrollable aggressive behaviors) and AE (weight increase) | (D) Olanzapine (Zyprexa®) 20 mg  (N) 1 | (D) Quetiapine (Seroquel®) (900 mg)  (N) 1 | (1) More likely to develop metabolic syndrome with olanzapine (HTR2C); quetiapine and ziprasidone were the only antipsychotics with standard response.  (2) CGI-S=7; CGI-I=2  (3) Yes |
| 8 | 14M | (M) Autistic Disorder (299.00) and symptoms of hyperactivity  (R) PCR (Severe motor hyperactivity and inattention, aggressiveness leading to hospitalization) | (D) Risperidone (Risperdal®) (1 mg)  (N) 1 | (D) - Long-acting methylphenidate (Equasym®) (30 mg)  Risperidone (Risperdal®) (3 mg)  (N) 2 | (1) Less likely to present extrapyramidal side effects with either risperidone or paliperidone (AKT1‐DDIT4‐FCHSD1‐RPTOR); more likely to respond to methylphenidate (ADRA2A, COMT).  (2) CGI-S=7; CGI-I=2  (3) NA |
| 9 | 13M | (M) - Autistic Disorder (299.00)  - Epilepsy (Lennox-Gastaut)  (R) PCR (heteroaggressive behavior) and AE (excessive sedation) | (D) - Rufinamide (Inovelon®) (400 mg)  - Risperidone (Risperdal®) (1 mg) - Valproic acid (Depakine Crono®) (1000 mg)  - Oxcarbazepine (Trileptal®) (1200 mg)  - Topiramate (Topamax®) (150 mg)  (N) 5 | (D) - Rufinamide (Inovelon®) (400 mg)  - Risperidone (Risperdal®) (2.5 mg) - Valproic acid (Depakine Crono®) (1000 mg)  - Oxcarbazepine (Trileptal®) (1200 mg)  (N) 4 | (1) More likely to respond to risperidone (DRD2, LOC730267, RGS4)  (2) CGI-S=6; CGI-I=2  (3) Yes |
| 10 | 16M | (M) - Moderate Mental Retardation (318.0)  - Prader-Willi syndrome  (R) PCR (Severe aggressiveness, exacerbation of repetitive behaviors, and depressive-like symptoms). | (D) - Topiramate (Topamax®) (200 mg)  - Risperidone (Risperdal®) (1.5 mg)  (N) 2 | (D) - Topiramate (Topamax®) (200 mg)  - Risperidone (Risperdal®) (6 mg) - Fluoxetine (40 mg)  (N) 3 | (1) Standard response to risperidone and topiramate; more likely to respond to fluoxetine (GSK3B).  (2) CGI-S=6; CGI-I= 2  (3) NA |

*PCR (Poor clinical response), AE (adverse events), CO (testing whether the current treatment was the best choice available)

**Table 2. Characteristics of the sample of children and adolescents living with parents.**

| **Case** | **Age (years)/ Sex** | **(M) Main diagnose(s) (DSM-IV)**  **(R) Reason for PGx testing (PCR/AE/CO)*** | **(D) pre-PGx testing Drugs (mg per day)**  **(N) Number of concomitant drugs** | **(D) post-PGx testing Drugs (mg per day)**  **(N) Number of concomitant drugs** | **(1) PGx testing recommendation**  **(2) Clinical outcome (CGI-S and CGI-I)**  **(3) Improvement of side effects** |
| --- | --- | --- | --- | --- | --- |
| 11 | 16F | (M) Organic Personality Disorder  (R) PCR (Mood instability, and recurrent self-harming and suicide attempts) | (D) - Venlafaxine (Vandral®) (225 mg)  - Lormetazepam (Noctamid®) (2 mg) - Diazepam (Valium®) (30 mg) - Aripiprazole (Abilify®) (15 mg)  (N) 4 | (D) - Fluoxetine (20 mg)  - Eslicarbazepine acetate (Zebinix®) (800 mg) - Lormetazepam (Noctamid®) (2 mg)  (N) 3 | (1) More likely to respond to fluoxetine (BDNF).  (2) CGI-S=6; CGI-I=2  (3) NA |
| 12 | 16F | (M) Bipolar I Disorder, most recent episode manic (severe with psychotic features, 296.44)  (R) PCR (risk of suicidal behavior) | (D) - Olanzapine (Zyprexa®) (20 mg) - Lithium (Plenur®) (400 mg) - Lorazepam (Orfidal®) (3 mg)  (N) 3 | (D) - Lithium (Plenur®) (800 mg)  - Quetiapine (Seroquel®) (100 mg)  (N) 2 | (1) More likely to respond to lithium (CACNG2); greater metabolization of olanzapine (CYP1A2); more likely to respond to paroxetine (HTR1A)  (2) CGI-S=7; CGI-I=1  (3) NA |
| 13 | 17F | (M) Major Depressive Episode, single episode (moderate; 296.22)  (R) PCR (resistant depressive symptoms after 4 months on fluoxetine 40 mg/day) | (D) Fluoxetine (40 mg)  (N) 1 | (D) Desvenlafaxine (Pristiq®) (50 mg)  (N) 1 | (1) More likely to respond either to paroxetine or desvenlafaxine (ABCB1); intermediate metabolizer of fluoxetine (CYP2D6)  (2) CGI-S=4; CGI-I=1  (3) NA |
| 14 | 15F | (M) ADHD, combined subtype (314.01)  (R) PCR (poor attention span) and AE (headaches) | (D) Atomoxetine (Strattera®) (60 mg)  (N) 1 | (D) Long-acting methylphenidate (Medikinet®) 20 mg  (N) 1 | (1) Poor metabolization of atomoxetine (CYP2D6); more likely to respond to methylphenidate (COMT, LPHN3)  (2) CGI-S=4; CGI-I=1  (3) Yes |
| 15 | 14F | (M) - Initial diagnosis of ADHD, combined subtype (314.01)  - Current diagnosis of Schizoaffective Disorder (295.70)  (R) PCR (Severe aggressiveness, repeated self-harming and suicide attempts, mandatory auditory hallucinations); and AE (akathisia) | (D) - Risperidone (Risperdal®) (1.5 mg) - Lisdexamfetamine (Elvanse®) (30 mg)  - Fluoxetine (40 mg)  (N) 3 | (D) - Lithium (Plenur®) (1000 mg)  - Quetiapine (Seroquel®) (750 mg)  (N) 2 | (1) More likely to display side effects and reduced response to fluoxetine (SLC6A4); increased risk of extrapyramidal side effects with risperidone (AKT1‐DDIT4‐FCHSD1‐RPTOR); more likely to respond to lithium (CACNG2); antipsychotics with standard response were olanzapine, quetiapine, and ziprasidone  (2) CGI-S=6; CGI-I=2  (3) Yes |
| 16 | 16M | (M) - Initial diagnosis of ADHD, combined subtype (314.01), and Asperger’s Disorder (299.80)  - Later on, developed  Schizophrenia, paranoid type (295.30)  (R) CO | (D) - Paliperidone (Invega®) (15 mg)  - Fluoxetine (40 mg)  - Lorazepam (Orfidal®) (1 mg) - Lisdexamfetamine (Elvanse®) (30 mg)  (N) 4 | (D) - Paliperidone (Invega®) (18 mg)  - Fluoxetine (40 mg) - Lisdexamfetamine (Elvanse®) (30 mg)  (N) 3 | (1) More likely to respond to both fluoxetine (BDNF) and less likely to display extrapyramidal symptoms  with paliperidone (AKT1‐DDIT4‐FCHSD1‐  RPTOR)  (2) CGI-S=4; CGI-I=4  (3) NA |
| 17 | 16F | (M) Major Depressive Episode, single episode (severe without psychotic features, 296.23)  (R) PCR (no response to escitalopram and severe suicidal ideation and insomnia); and AE (increased weight with olanzapine, and akathisia and neck dystonia with risperidone) | (D) - Chlordiazepoxide (Tranxilium®) (10 mg)  - Escitalopram (Heipram®) (20 mg) - Olanzapine (Zyprexa®) (10 mg)  (N) 3 | (D) - Lithium (Plenur®) 1200 mg  - Fluvoxamine (Dumirox®) (200 mg)  (N) 2 | (1) More likely to respond to fluvoxamine (BDNF, HTR1A); high risk for extrapyramidal symptoms with paliperidone (AKT1‐DDIT4‐FCHSD1‐RPTOR); standard response to escitalopram and olanzapine.  (2) CGI-S=5; CGI-I=1  (3) Yes |
| 18 | 16M | (M) Major Depressive Episode, single episode (severe without psychotic features, 296.23)  (R) CO | (D) - Fluoxetine (20 mg)  - Lithium (Plenur®) (1200 mg)  (N) 2 | (D) - Fluoxetine (20 mg)  - Lithium (Plenur®) (800 mg)  (N) 2 | (1) More likely to respond (BDNF) and to be an intermediate metabolizer of fluoxetine (CYP2D6)  (2) CGI-S=5; CGI-I=3  (3) NA |
| 19 | 13M | (M) - Tourette’s Syndrome (307.23)  - ADHD, combined subtype (314.01)  - Obsessive-Compulsive Disorder (300.3); PANDAS positive  (R) PCR (severe obsessions and suicidal ideation) | (D) - Sertraline (Besitran®) (100 mg)  - Long-acting methylphenidate (Medikinet®) (40 mg)  (N) 2 | (D) - Sertraline (Besitran®) (150 mg)  - Long-acting methylphenidate (Medikinet®) 40 mg  - Quetiapine (Seroquel®) (50 mg)  (N) 3 | (1) More likely to respond either to citalopram, escitalopram, or desvenlafaxine (ABCB1); standard response to sertraline and quetiapine  (2) CGI-S=5; CGI-I=2  (3) NA |
| 20 | 13F | (M) - Major Depressive Disorder, single episode (in full remission, 296.26)  - ADHD, combined subtype (314.01)  (R) CO; and AE (Neck dystonia with low doses of paliperidone) | (D) - Fluoxetine (20 mg)  - Long-acting methylphenidate (Medikinet®) 20 mg  - Paliperidone (Invega®) (3 mg)  (N) 3 | (D) - Long-acting methylphenidate (Medikinet®) 50 mg  (N) 1 | (1) More likely to respond (BDNF) and to be an intermediate metabolizer (CYPD26) of fluoxetine; more likely to respond to methylphenidate (COMT,  LPHN3) and to develop extrapyramidal symptoms with paliperidone (AKT1-DDIT4-FCHSD1-RPTOR)  (2) CGI-S=4; CGI-I=2  (3) Yes |

*PCR (Poor clinical response), AE (adverse events), CO (testing whether the current treatment was the best choice available)
